# Single-Dose Longitudinal Pharmacokinetic Evaluation of Doravirine in Pregnant Women Living With HIV: Protocol for a Phase 1 Study

**DOI:** 10.2196/89990

**Published:** 2026-07-10

**Authors:** Amanda Poliseno, Lisa Rahangdale, Cynthia Gay, Emma Trawick Roberts, Angela Kashuba

**Affiliations:** 1 Division of Pharmacotherapy and Experimental Therapeutics, Eshelman School of Pharmacy, University of North Carolina at Chapel Hill, 120 Mason Farm Road, Chapel Hill, NC, 27599, United States, 1 919-962-5344; 2 Department of Obstetrics and Gynecology, University of North Carolina at Chapel Hill, Chapel Hill, NC, United States; 3 Division of Infectious Diseases, UNC School of Medicine, University of North Carolina at Chapel Hill, Chapel Hill, NC, United States

**Keywords:** HIV, AIDS, pregnancy, doravirine, pharmacokinetics, postpartum

## Abstract

**Background:**

Women are largely underrepresented in clinical trials of antiretroviral therapy, constituting approximately 20% of participants. Trials often exclude pregnant women from participation to minimize fetal risk, and women enrolled in clinical trials who have a positive pregnancy test may be discontinued from the study. This results in an average 7-year lag between the time of drug approval and when dosing information for pregnancy is available. However, there may be low-risk opportunities to obtain dosing information earlier for pregnant women who need treatment with newer medications. We demonstrate this with a clinical trial of doravirine, a Food and Drug Administration (FDA)–approved nonnucleoside reverse transcriptase inhibitor, designed to assess the pharmacokinetics and dosing strategy for doravirine during pregnancy while maintaining safety for participants.

**Objective:**

The primary objective of this study is to quantify single-dose total and protein-unbound pharmacokinetics of doravirine in blood plasma from women living with HIV during their second trimester, third trimester, and postpartum periods. The secondary objective is to evaluate the safety and tolerability of single doses of doravirine in pregnant participants living with HIV.

**Methods:**

The proposed study is a single-site phase 1 evaluation of single doses of doravirine across the gestational period in pregnant female participants (n=10) living with HIV currently controlled with antiretroviral therapy. Single doses were administered in the second and third trimesters and postpartum. Pharmacokinetic sampling occurred over a 72-hour dosing interval, and total and protein-unbound drug concentrations were assessed across the 3 dosing phases using LC-MS/MS methods validated according to FDA criteria. Pharmacokinetic parameters of peak drug concentration, concentration at 12 hours into the dosing interval, concentration at the end of the dosing interval, time to reach maximum concentration, and area under the curve were estimated using noncompartmental linear up-log down methods (version 8.6; Phoenix WinNonlin). Comparisons across the 3 sampling visits were made using the Friedman test and post hoc Wilcoxon signed-rank tests with Bonferroni adjustment (version 13.0; SigmaPlot).

**Results:**

The proposed study was funded in 2020 and approved by the University of North Carolina-Chapel Hill institutional review board on March 9, 2021. Participant recruitment began the same day, and the first participant was enrolled on June 9, 2022. The last participant was enrolled on October 3, 2024, and all participants were off the study on April 8, 2025. In total, 7 participants were enrolled in the study, and 5 were evaluable.

**Conclusions:**

The data collected with this novel single-dose pharmacokinetic study design will provide important insights into the clinical management of patients on doravirine during pregnancy and provide an effective model for obtaining early pharmacokinetic and dosing information for new drugs that can be used in pregnant women.

## Introduction

### Background

At any given time globally, more than 1 million women living with HIV are pregnant [1]. Pregnant women need safe and effective therapy for the treatment of HIV infection for their health and the health of their babies but are the least likely to have timely data regarding optimal drug dosing and efficacy when a drug is marketed [2]. Drug trials often exclude pregnant women to minimize fetal risk. For clinical trials of antiretroviral therapy (ART), women are largely underrepresented, constituting approximately 20% of participants [3]. These exclusions limit available ART pharmacokinetic data and, therefore, evidence-based dosing recommendations during pregnancy.

Extensive physiologic changes occur during pregnancy, which can alter absorption, distribution, metabolism, and elimination of ART [4], and often do not return to baseline until 2 to 12 weeks postpartum [4]. More specifically, pregnancy has been found to decrease drug absorption through delayed gastric motility and emptying, increase plasma volume and decrease plasma protein binding, increase the expression of certain hepatic drug-metabolizing enzymes including CYP3A4, increase renal clearance through increased glomerular filtration, and increase hepatic clearance through increased hepatic blood flow [4]. All of these changes have the potential to lead to lower drug exposure during pregnancy [4].

Understanding how pregnancy-related physiologic changes affect ART pharmacokinetics is vital to ensuring women receive appropriate dosing to maximize efficacy and minimize the development of viral drug resistance. However, excluding pregnant women from pre–new drug application clinical trials leads to gaps in drug dosing information in the product label and substantial delays before dosing information becomes available to pregnant women [5].

The 21st Century Cures Act established the Task Force on Research Specific to Pregnant and Lactating Women [6]. In September 2018, this committee presented its report to the Secretary of Health and Human Services and Congress, outlining strategies for identifying and addressing knowledge gaps regarding drug use during pregnancy. Recently, a *New England Journal of Medicine* Perspective article also discussed several approaches to obtaining efficacy, safety, and pharmacokinetic information in pregnancy [7], including staggered trial designs, embedded trial designs, and opportunistic study designs.

Nonnucleoside reverse transcriptase inhibitors (NNRTIs) have been used to treat HIV infection for decades, and a high placental transfer helps effectively prevent vertical HIV transmission [4]. Doravirine is a Food and Drug Administration (FDA)–approved NNRTI used to treat HIV infection with a unique resistance and safety profile [8] that could be used effectively in pregnancy. Doravirine (tradename PIFELTRO) and doravirine combined with lamivudine and tenofovir disoproxil fumarate (tradename DELSTRIGO) were approved in the United States, the European Union, Canada, and Australia in 2018 for the treatment of HIV-1 infection in adults [8]. The antiviral activity of doravirine against wild-type virus (EC_50_=12 nM) is also more potent than that of first-generation NNRTIs such as efavirenz (EC_50_=30 nM) [8].

Doravirine can be transferred to the fetus. In animal embryo-fetal studies, doravirine fetal plasma concentrations at gestation day 20 were up to 40% (rabbits) and 52% (rats) of maternal concentrations. However, in rat and rabbit reproduction studies, no adverse developmental effects were observed when doravirine was administered at exposures ≥8 times the exposure in humans at the recommended dose [8]. At the time of protocol writing, no exposures to doravirine were noted in the antiretroviral pregnancy registry. In the most recent pregnancy registry interim report [9], 24 live births were noted in women exposed to doravirine, with 2 of 20 live births exposed during the first trimester characterized as having defects according to the Centers for Disease Control and Prevention criteria. More data are needed to determine whether the risk of doravirine is above the baseline risk of other ARTs.

Doravirine is predominantly metabolized by CYP3A enzymes [8]. While coadministration of doravirine with drugs that induce or inhibit CYP3A may alter its plasma concentrations, doravirine is unlikely to have a clinically relevant effect on the concentrations of other CYP3A substrates [8]. Pregnancy itself can significantly increase the clearance of CYP3A substrates, as well as decrease total drug concentrations (due to increases in body water) and increase protein-unbound concentrations (due to decreases in drug-binding proteins such as albumin) [4]. Because there is significant potential for pregnancy to alter the pharmacokinetics of doravirine, this study evaluated the changes in pharmacokinetics in the second and third trimester, compared with postpartum conditions. Because the preclinical reproductive toxicity data for doravirine showed minimal risk to maternal and fetal health [8], doravirine was a good candidate for a small, controlled pharmacokinetics and safety trial in pregnancy.

At the time of study initiation, no human pharmacokinetic data had been generated for doravirine in pregnancy. This study aimed to close this knowledge gap using a safe and efficient trial design in pregnant women living with HIV. Because the first trimester is the most critical period of fetal growth and development [3], this study was designed to administer the drug after organogenesis was complete [3]. Single doses of doravirine were administered in the second and third trimester and again postpartum. Pharmacokinetic sampling occurred up to 72 hours after dosing, and total and protein-unbound drug concentrations and pharmacokinetic parameters were compared across these 3 dosing phases.

### Objectives

The primary objective of this study was to describe single-dose total and protein-unbound pharmacokinetics of doravirine in the blood plasma of women living with HIV during their second trimester, third trimester, and postpartum periods. The secondary objective of this study was to evaluate the safety and tolerability of single doses of doravirine in pregnant participants living with HIV.

## Methods

### Recruitment

The proposed study was approved by the local institutional review board (IRB) on March 9, 2021. Participant recruitment began the same day, and the first participant was enrolled on June 9, 2022. The last participant was enrolled on October 3, 2024. Recruitment ended December 2, 2024. All study participants were off study on April 8, 2025. This study planned to enroll 10 participants at a single United States site, the University of North Carolina (UNC) Infectious Diseases Clinic, a hospital-based outpatient clinic in Chapel Hill, North Carolina. Data collection occurred in the UNC Infectious Diseases Clinic, the Clinical & Translational Research Center, and other areas of the UNC-Chapel Hill campus such as the UNC obstetrical clinics.

Of all people living with HIV who attend the UNC Infectious Diseases Clinic, 95% had consented to having their participant information available in a secure clinic database to identify their eligibility for open research studies and to being notified of those studies (as per UNC IRB 99-MED-408). The Infectious Diseases Clinic has a full-time research screener to assess participant eligibility for open research projects. This study used this IRB-approved screening and recruitment process, whereby the clinic screener prescreened participants in the clinic database and alerted research staff to potentially eligible participants [10]. Specific recruitment methods used (phone, email, or in person) for any individual participant depended on the methods for which that participant previously provided permission. For participants who could not be contacted by phone and/or email after a maximum of 3 attempts, research staff approached them in the clinic waiting room (if the patient has given permission to do so) on the day of their visit to notify them that they may be eligible for a study. Those who were interested were asked to move to a private room in the UNC Infectious Diseases Clinic or research clinic to undergo further screening and, if eligible and interested, to provide informed consent. IRB-approved culturally appropriate flyers were also posted in designated areas of the clinics for the duration of study enrollment with information about the name and overarching purpose of the study.

The target study population often faced additional logistical hurdles to participation in this research, such as reliable transportation, distance to study locations, and childcare postpartum. Transportation and lodging accommodations were provided for participants if needed. When returning for the postpartum sampling visits, participants were provided with options to heat formula and a room for the infants to stay safely.

### Study Design

**Figure 1.**
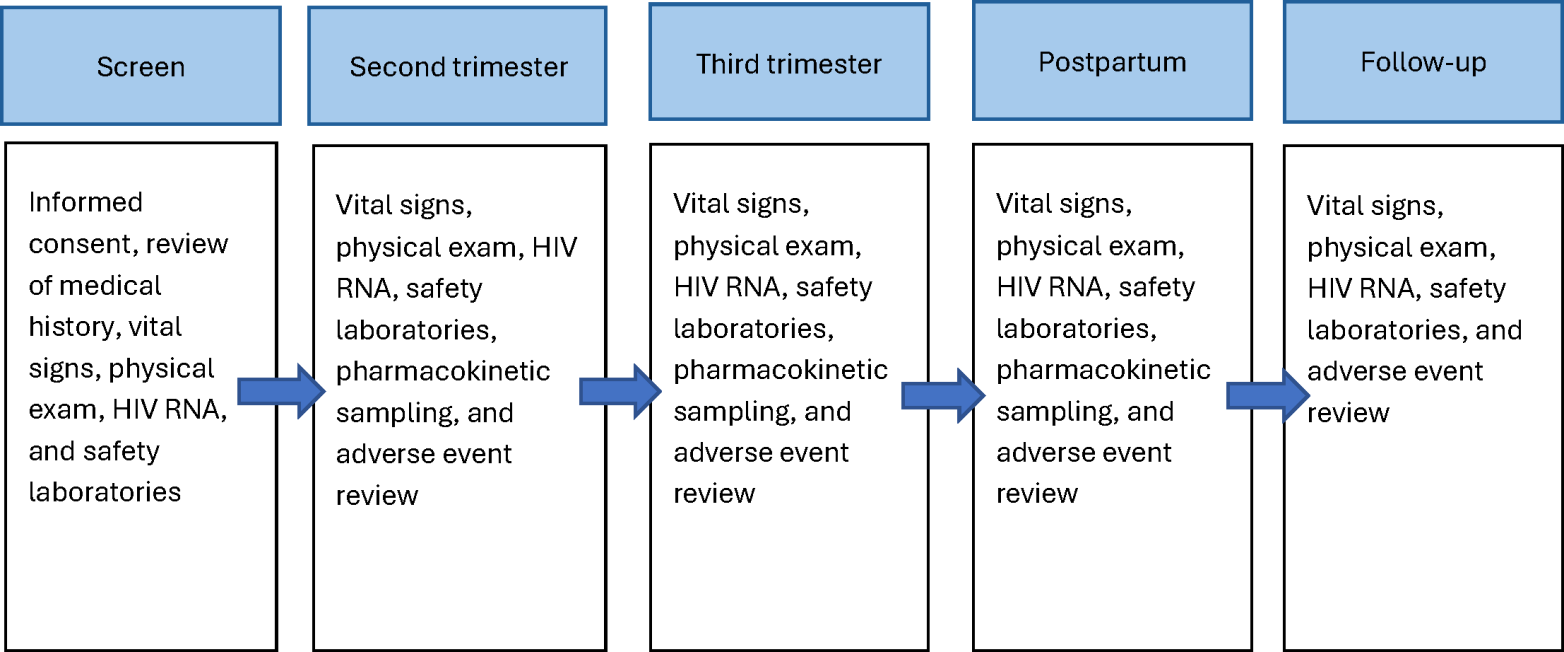
Study schema for institutional review board (20‐0052).

Eligible participants were referred to study staff for prescreening using a standardized IRB-approved prescreening questionnaire either in person in the clinic or over the phone. This questionnaire was used to confirm a participant’s age, HIV and pregnancy status, current medications, and willingness to participate in study activities. Participants who were deemed eligible via the prescreen were scheduled for an in-person screening visit in the research clinic. This visit was scheduled within 28 days prior to the anticipated second trimester enrollment, as outlined in the study schema (Figure 1). All visits were conducted in the Clinical & Translational Research Center at UNC-Chapel Hill.

Upon arrival at the screening visit, all participants reviewed the Health Insurance Portability and Accountability Act and informed consent forms with a study team member. Standard operating procedures were followed for the consent process according to the standards set forth in the International Council for Harmonisation Good Clinical Practice guidelines [11]. If a participant provided consent, the screening visit commenced. The study team obtained and recorded the participants’ medical, medication, and obstetrical histories. A physical examination was performed, and vital signs were assessed. The study team reviewed documentation of the obstetrical ultrasound performed as part of standardized obstetrical care to document a healthy single intrauterine gestation. Safety laboratory values were obtained to assess major organ function as noted in the schedule of events (Table 1). Documentation of an undetectable HIV RNA was obtained for the prior 90 days.

**Table 1.** Schedule of events for institutional review board (20‐0052).

Activity	Screen (−28 to 0 d)	Second trimester (20‐26 wk)	Third trimester (30‐34 wk)	Postpartum (4‐8 wk after delivery)	Follow-up (within 14 d after last sample)
Informed consent	✓				
Review of medical history	✓				
Vital signs^a^	✓	✓	✓	✓	✓
Physical examination	✓	✓	✓	✓	✓
HIV RNA	✓	✓	✓	✓	✓
Infant HIV DNA^b^				✓	
Safety laboratory values^c^	✓	✓	✓	✓	✓
Pharmacokinetic sampling^d^		✓	✓	✓	
Adverse event review		✓	✓	✓	✓

a Vital signs include blood pressure, pulse, respiratory rate, temperature, and weight. Height should be documented at the screening visit.

b Infant HIV DNA data will be obtained from electronic medical records.

c Safety laboratory values include complete blood count with differential and the following serum chemistries: sodium, potassium, chloride, carbon dioxide, blood urea nitrogen, creatinine, glucose, calcium, albumin, total protein, aspartate aminotransferase, alanine aminotransferase, alkaline phosphatase, total bilirubin, total cholesterol, triglycerides, high-density lipoprotein, low-density lipoprotein, cholesterol, and lipase. Urinalysis will be completed if it has not been done as part of obstetrical care during the previous month.

d As outlined in the Study Design section, pharmacokinetic samples for total blood plasma concentrations will be obtained at 0, 0.5, 1, 2, 4, 6, 8, 10, 12, and 24 hours around an observed dose of doravirine during all pharmacokinetic visits. Samples will be collected at 48 and 72 hours after the observed dose for participants who are able to return to the clinic.

Study participants were included if they were aged at least 18 years, living with HIV, willing and able to comply with scheduled visits and trial procedures, had been on stable combination ART for at least 30 days prior to trial enrollment, had documentation of plasma HIV RNA of <50 copies/mL within 90 days prior to enrollment, had aspartate aminotransferase and alanine aminotransferase levels <3 times the upper limit of normal, and had a hemoglobin level lower than Division of AIDS (DAIDs) grade 2 (9.0 g/dL) [12]. Participants were excluded if they had multiple gestations, active opportunistic infections, or any current obstetrical complications that would deem them unsuitable for study participation; had evidence of fetal anomalies in their current pregnancy, renal or hepatic impairment, or a significant illness or condition at the time of enrollment that would interfere with or serve as a contraindication to protocol adherence or safety assessment; had pregnancies that became complicated or active hepatitis C infection; had clinically significant laboratory values >DAIDs grade 2; or were receiving CYP3A inducers or moderate to strong CYP3A inhibitors (Multimedia Appendix 1). For participants who passed initial screening, subsequent study visits were scheduled. The second trimester visits were scheduled within a window of 20 to 26 weeks’ gestation, third trimester visits were scheduled within 30 to 34 weeks’ gestation, and postpartum visits were scheduled within 4 to 8 weeks of delivery.

Upon admission to the first pharmacokinetics visit, initial vital signs and laboratory safety tests were obtained to verify participant safety (Table 1). If the participant was deemed still eligible, they were administered a single 100 mg dose of doravirine witnessed by study staff. Standardized breakfast options created by registered dietitians were ordered for the participant to eat with their doravirine dose (Multimedia Appendix 2). Blood plasma sampling occurred at the following time points at and after the observed dose: 0, 0.5, 1, 2, 4, 6, 8, 10, 12, 24, 48, and 72 hours. Total blood volume for the study samples was approximately 30 mL (2 tablespoons). Participants were provided meals and snacks throughout the duration of the visit. Review of an adverse events questionnaire was completed preceding discharge. Obstetrical standard-of-care evaluations were completed with the participants’ regular care providers.

Within 14 business days following completion of all pharmacokinetics visits, all participants returned to clinic for a final safety assessment. Vital signs and physical examinations were conducted. Documentation of maternal outcomes was completed, as well as that for the neonate (including HIV DNA, infant outcomes at delivery, and Apgar scores).

Adverse events were assessed using a standardized checklist (Multimedia Appendix 3). If a participant answered “yes” to any of the items on the checklist, additional probing was conducted by study staff to determine the nature of the adverse event. Any reported adverse event was documented, causality was determined, the event was graded using the DAIDs adverse event grading table [12], and the event was followed to resolution. Participants were considered off study when study activities were completed and all adverse events had been followed to completion or resolution.

### Ethical Considerations

This study received ethics approval from the UNC-Chapel Hill IRB (reference ID 453015).

The investigators obtained informed consent from each participant before starting any study procedures according to the standards set forth in the International Council for Harmonisation Good Clinical Practice guidelines [11] and per unit standard operating procedures. This involved reading over the IRB-approved consent form with the participant in a private space, soliciting questions from the participant, allowing the participant ample time alone to review the form, soliciting questions again, and then offering the participant the opportunity to sign the consent form. To ensure understanding, study staff asked questions of the participants regarding study procedures. Participants who could not demonstrate adequate understanding of key concepts after educational efforts were not enrolled in the study.

Participants were compensated for all parts of the study completed. Payment was provided in the form of Visa gift cards, and the amount per activity was reviewed and approved by the local IRB. Participants received US $50 for completing the screening visit, US $275.00 for each completed sampling visit, US $25.00 for each blood draw at the 24-, 48-, and 72-hour time points at each sampling visit, and US $50 for completion of the follow-up visit. Participants were provided standardized breakfasts, additional meals and snacks at each sampling visit, and parking tokens at every visit. Transportation and lodging were also provided to participants if needed.

Confidentiality was maintained by storing all specimens for current and future use with a unique identifying number, which was linked to the participant’s name, social security number, address, telephone number, and hospital medical record number. The principal investigators and study staff were the only people with access to the identifying information. Any information provided to other people working on this study was through the study ID number, and no other identifying information. The records were secured in a locked file cabinet in a locked room in a badge access only office suite of the principal investigator. All electronic data for this study were stored on a dedicated university server that contained extensive protections and securities.

Participant confidentiality and privacy were strictly held in trust by the participating investigators, their staff, and the sponsors and their representatives. This confidentiality was extended to the testing of biological samples in addition to the clinical information relating to participants. Therefore, the study protocol, documentation, data, and all other information generated was held in strict confidence. No information concerning the study or the data was released to any unauthorized third party without prior written approval of the sponsor. The study monitor, other authorized representatives of the sponsor, representatives of the IRB, and regulatory agencies were able to inspect all documents and records required to be maintained by the investigator, including but not limited to, medical records (office, clinic, or hospital) and pharmacy records for the participants in this study. The clinical study site permitted access to such records. The study participant’s contact information was securely stored at each clinical site for internal use during the study. At the end of the study, all records are kept in a secure location for at least 5 years, as specified by the sponsor.

### Statistical Analysis

Noncompartmental pharmacokinetic analysis was performed to calculate pharmacokinetic parameters using Phoenix WinNonlin or NLME (version 8.5; Certara). Summary statistics were generated for these pharmacokinetic parameters from the 3 visits. Nonparametric statistical testing was used to identify significant differences between the visits for both clinical data and pharmacokinetic data.

Due to standard parametric approaches being unsuitable for this small sample size, the Friedman test was used, with subsequent pair-wise comparisons using the Wilcoxon signed-rank tests with Bonferroni correction, to determine differences between the pharmacokinetic phases for both total and protein-unbound pharmacokinetic parameters (SigmaPlot, version 13.0; Systat Software Inc).

### Variables and Time Points of Interest

Primary variables of interest include participant demographics and blood plasma drug concentrations. Pharmacokinetic sampling in blood plasma was completed on study visits during the second and third trimesters, as well as postpartum at the following time points: 0, 0.5, 1, 1.5, 2, 3, 4, 5, 6, 8, 10, 12, 24, 48, and 72 hours after dosing. Pharmacokinetic parameters of particular interest were calculated from these data (Phoenix WinNonlin or NLME) including peak drug concentration, concentration at 12 hours into the dosing interval, concentration at the end of the dosing interval, area under the concentration-time curve over 24 hours, time to reach maximum concentration, and terminal elimination rate.

### Sample Size Determination

Doravirine has modest interparticipant variability in pharmacokinetic parameters in healthy volunteers (approximately 23%‐35% with peak drug concentration and concentration at the end of the dosing interval, respectively) [13], and our LC-MS/MS methods have low assay variability (assay precision 3%‐11%) [14]. Therefore, the proposed sample size (n=10) was identified pragmatically to generate robust pharmacokinetic data adequate for understanding blood plasma pharmacokinetics of doravirine in pregnant women living with HIV. Although the resulting pharmacokinetic estimates are not as precise as those obtained with a larger sample size, the repeated measures approach with comprehensive pharmacokinetic sampling provides reasonable estimates of central tendency to inform future clinical studies. Our experience with previous pregnancy pharmacokinetic investigations suggested that this sample size will provide us with reasonable estimates of drug exposure [15].

### Sample Analysis

Whole blood collected at each time point was processed within 30 minutes of collection (or up to 1 h on ice) by centrifugation at 3000 rpm at 4 °C for 10 minutes to separate plasma. The plasma was then aliquoted into appropriately labeled 1.2 mL Corning cryogenic vials and stored in a continuously monitored freezer (Minus80 Monitoring) at –80 ℃ until analysis.

Total and protein-unbound doravirine concentrations were measured using LC-MS/MS assays validated according to FDA criteria [16] with lower limits of quantification of 3 ng/mL and 0.3 ng/mL, respectively. Interassay precision and accuracy were within the +15% and –15% (+20% and –20% lower limit of quantification) acceptance criteria. Further analytical method details will be discussed in the main results paper.

## Results

The proposed study was funded in 2020 and approved by the UNC-Chapel Hill institutional review board on March 9, 2021. In total, 7 participants were enrolled in the study and 5 were found to be evaluable. One participant was withdrawn from the study due to viral rebound between the second and third trimester visits; upon assessment, this rebound was deemed unrelated to the study product. One participant voluntarily withdrew from the study before the postpartum sampling visit due to scheduling conflicts after birth.

The proposed study allowed participants to self-identify their race and ethnicity. Of the evaluable participants, 60% (n=3) reported their ethnicity as Hispanic and 40% (n=2) reported their ethnicity as non-Hispanic. Of the evaluable participants, 60% (n=3) reported their race as White, 20% (n=1) reported their race as African American, and 20% (n=1) reported their race as other (Hispanic). The mean age of evaluable participants in this study was 26.6 years (SD 3.07) with a median of 28 (range 21-30) years. The mean BMI of evaluable participants in this study was 36.43 kg/m^2^ (SD 7.4) with a median of 36.72 (range 26.37‐49) kg/m^2^. No adverse events were reported by any participants in this study. No adverse maternal or fetal outcomes were reported from any participant in this study. Data analysis took place through 2025, and the manuscript with the pharmacokinetic data will be published in 2026.

## Discussion

### Expected Findings

This novel single-dose pharmacokinetics approach is anticipated to provide a safe opportunity to study the pharmacokinetics of medications earlier after drug approval. The successful completion of this feasibility study allows for further use of this approach. The data generated will inform better clinical care for women. Because doravirine is a CYP3A substrate, it is anticipated that pregnancy-related changes in CYP3A activity will lower plasma concentrations during pregnancy. Because doravirine is modestly protein bound [8], no changes in protein binding during pregnancy are anticipated. Because doravirine appears to have a wide therapeutic index [16], the extent to which total and protein-unbound drug concentrations change will dictate whether a dose adjustment may be necessary in the second or third trimesters of pregnancy.

### Limitations

Identifying and enrolling eligible pregnant women living with HIV into pharmacokinetic clinical trials is challenging. At our site, 52 HIV-positive pregnancies were reported during the time of this study’s enrollment. Of the 52 pregnancies, 1 (2%) was ineligible due to the participant being younger than 18 years, 1 (2%) was ineligible due to incarceration, 3 (6%) were ineligible due to concomitant interacting medications (cobicistat), 5 (10%) were ineligible due to termination of pregnancy or miscarriage, 6 (12%) were unable to be contacted about the study, and 6 (12%) were presented the study but did not want to participate. When the study started recruiting, 8 (15%) patients were already in their second or third trimester. During study recruitment, 8 (15%) patients entered care in their third trimester, and 6 (12%) participants were ineligible due to language barriers. Eight (15%) of the 52 pregnancies were screened for eligibility. One (2%) woman did not attend her enrollment visit and was lost to follow-up. Of the 7 (14%) participants enrolled in the study, 5 (10%) successfully completed all portions.

The limited sample size limits our ability to detect statistically significant differences in the pharmacokinetic parameters generated. However, the repeated measures in these women improve the robustness of our data. Additionally, the calculation of pharmacokinetic parameters allows us to determine at each visit the proportion of women who have drug exposure that may be cause for concern.

### Conclusions

The data collected with this novel single-dose pharmacokinetic study design are anticipated to provide important insights into the clinical management of patients on doravirine during pregnancy and provide an effective model for obtaining early pharmacokinetic and dosing information for new drugs that will be used in pregnant women. To date, no other single-dose pharmacokinetic studies have been conducted in the pregnancy setting.

## Supplementary material

10.2196/89990 Multimedia Appendix 1 CYP3A inducers and inhibitors excluded from trial.

10.2196/89990 Multimedia Appendix 2 Standardized breakfast options.

10.2196/89990 Multimedia Appendix 3 Adverse event checklist for institutional review board (20-0052).

10.2196/89990 Checklist 1 CONSORT checklist.

## Abbreviations

ART: antiretroviral therapy
DAIDS: Division of AIDS
FDA: Food and Drug Administration
IRB: institutional review board
NNRTI: nonnucleoside reverse transcriptase inhibitor
UNC: University of North Carolina

## References

[1] HIV - prevention of mother-to-child transmission. World Health Organization.

[2] Lyerly AD, Beigi R, Bekker LG (2021). Ending the evidence gap for pregnancy, HIV and co-infections: ethics guidance from the PHASES project. J Int AIDS Soc.

[3] Fairlie L, Waitt C, Lockman S (2019). Inclusion of pregnant women in antiretroviral drug research: what is needed to move forwards?. J Int AIDS Soc.

[4] Briceño-Patiño N, Prieto MC, Manrique P, Calderon-Ospina CA, Gómez L (2025). Pharmacokinetic adaptations in pregnancy: implications for optimizing antiretroviral therapy in HIV-positive women. Pharmaceutics.

[5] Colbers A, Mirochnick M, Schalkwijk S, Penazzato M, Townsend C, Burger D (2019). Importance of prospective studies in pregnant and breastfeeding women living with human immunodeficiency virus. Clin Infect Dis.

[6] (2018). Task force on research specific to pregnant women and lactating women: report to Secretary, Health and Human Services Congress. https://www.nichd.nih.gov/sites/default/files/2018-09/PRGLAC_Report.pdf.

[7] Eke AC, Dooley KE, Sheffield JS (2019). Pharmacologic research in pregnant women - time to get it right. N Engl J Med.

[8] (2025). DELSTRIGO® (doravirine, lamivudine, and tenofovir disoproxil fumarate) tablets, for oral use. Merck & Co.

[9] (2025). Antiretroviral pregnancy registry interim report for 1 January 1989 through 31 July 2025. https://www.apregistry.com/forms/interim_report.pdf.

[10] Poliseno A, Ferguson E, Perry R (2023). Establishing novel antiretroviral imaging for hair to elucidate nonadherence: protocol for a single-arm cross-sectional study. JMIR Res Protoc.

[11] (2025). ICH harmonised guideline: guideline for good clinical practice E6(R3). https://database.ich.org/sites/default/files/ICH_E6%28R3%29_Step4_FinalGuideline_2025_0106.pdf.

[12] (2017). Division of AIDS (DAIDS) table for grading the severity of adult and pediatric adverse events. https://rsc.niaid.nih.gov/sites/default/files/daidsgradingcorrectedv21.pdf.

[13] Anderson MS, Gilmartin J, Cilissen C (2015). Safety, tolerability and pharmacokinetics of doravirine, a novel HIV non-nucleoside reverse transcriptase inhibitor, after single and multiple doses in healthy subjects. Antivir Ther.

[14] Schauer AP, Sykes C, Cottrell ML, Imaz A, Podzamczer D, Kashuba AD (2022). Validation of an LC-MS/MS assay for the simultaneous determination of bictegravir, doravirine, and raltegravir in human plasma. J Pharm Biomed Anal.

[15] Patterson KB, Dumond JB, Prince HA (2013). Protein binding of lopinavir and ritonavir during 4 phases of pregnancy: implications for treatment guidelines. J Acquir Immune Defic Syndr.

[16] Yee KL, Ouerdani A, Claussen A, de Greef R, Wenning L (2019). Population pharmacokinetics of doravirine and exposure-response analysis in individuals with HIV-1. Antimicrob Agents Chemother.

