# Supplementary material for: Single-Dose Longitudinal Pharmacokinetic Evaluation of Doravirine in Pregnant Women Living With HIV: Protocol for a Phase 1 Study
Source: JMIR Res Protoc. 2026 Jul 10;15:e89990. doi: 10.2196/89990 (PMC13352967; doi:10.2196/89990)
Supplement: Multimedia Appendix 1 [file resprot-v15-e89990-s001.docx]

CYP3A inducers and inhibitors excluded from trial.

| **CYP3A Inducers** | **CYP3A Inhibitors** |
| --- | --- |
| • oxcarbazepine  • carbamazepine  • phenobarbital  • phenytoin  • enzalutamide  • St. John’s wort  • rifampin  • rifapentine  • mitotane  • atazanavir  • maraviroc  • darunavir  • ritonavir  • efavirenz  • tipranavir   - etravirine - nevirapine | • clarithromycin  • boceprevir  • cobicistat  • danoprevir and ritonavir  • elvitegravir and ritonavir  • indinavir and ritonavir  • itraconazole  • ketoconazole  • lopinavir and ritonavir  • paritaprevir and ritonavir  • posaconazole  • ritonavir  • saquinavir and ritonavir  • telaprevir  • tipranavir and ritonavir  • grapefruit juice  • idelasib  • nefazodone  • nelfinavir |
