# Supplementary material for: Single-Dose Longitudinal Pharmacokinetic Evaluation of Doravirine in Pregnant Women Living With HIV: Protocol for a Phase 1 Study
Source: JMIR Res Protoc. 2026 Jul 10;15:e89990. doi: 10.2196/89990 (PMC13352967; doi:10.2196/89990)
Supplement: Multimedia Appendix 2 [file resprot-v15-e89990-s002.docx]

Supplement 2: Standardized Breakfast Options

For this study, you will need to consume a standardized breakfast on the days you receive the study medication. Please choose one option from the items listed below so we can have it ready for you the morning of your appointment.

| **Breakfast Sandwich**  Fried egg (1)  Cheddar cheese (1 slice)  Turkey bacon (2 slices)  Wheat bread, white bread (2 slices) or English Muffin  Banana (1)  Orange juice (1) |
| --- |
|  |
| **Eggs and Pancakes**  Pancakes (2) with syrup packet (1)  Scrambled eggs (standard serving is ½ cup)  Fresh fruit cup (1) |
|  |
| **Cereal**  Granola cereal (1 box)  Whole milk (1 carton)  Fresh fruit cup (1)  Hard-boiled egg (1) |
|  |
| **Bagel**  Plain bagel (1) (request toasted or untoasted)  Peanut butter (1)  Activia Vanilla yogurt (1) |
|  |
| **Gluten Free Option**  Gluten free bread (2 slices)  Peanut butter (1)  Strawberry Greek yogurt (1) |
