# Supplementary figures and images for: Single-Dose Longitudinal Pharmacokinetic Evaluation of Doravirine in Pregnant Women Living With HIV: Protocol for a Phase 1 Study
Source: JMIR Res Protoc. 2026 Jul 10;15:e89990. doi: 10.2196/89990 (PMC13352967; doi:10.2196/89990)

Adverse event checklist for IRB 20-0052.


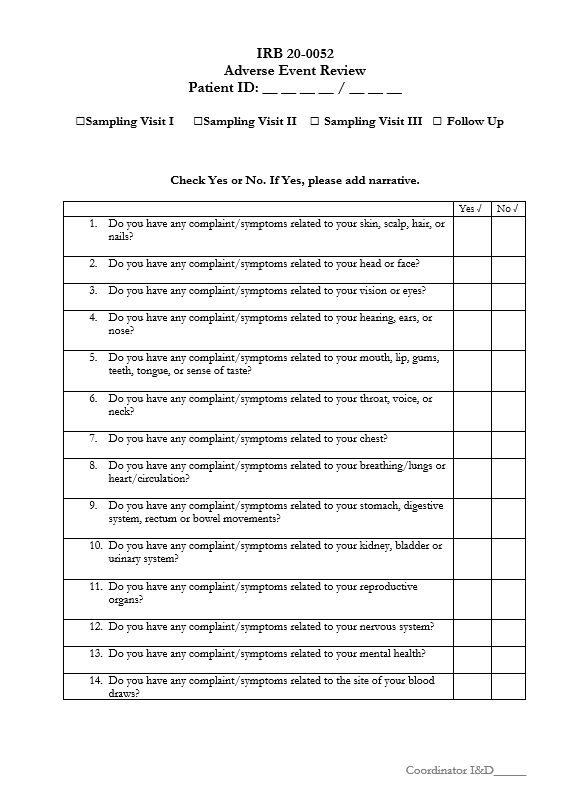

Supplement: Multimedia Appendix 3 [file resprot-v15-e89990-s003.docx]
